# The advertisement call and comments on the distribution of *Eleutherodactylus bilineatus* Bokermann, 1975, an endemic frog of Bahia State, Brazil (Amphibia, Anura)

**DOI:** 10.3897/zookeys.677.12309

**Published:** 2017-05-30

**Authors:** Iuri Ribeiro Dias, Caio Vinicius de Mira-Mendes, Carlos Augusto Souza-Costa, Flora Acuña Juncá, Mirco Solé

**Affiliations:** 1 Departamento de Ciências Biológicas, Universidade Estadual de Santa Cruz, Rodovia Jorge Amado, km 16, 45662-900 Ilhéus, Bahia, Brazil; 2 Programa de Pós-Graduação em Zoologia, Universidade Estadual de Santa Cruz, Rodovia Jorge Amado, km 16, 45662-900 Ilhéus, Bahia, Brazil; 3 Programa de Pós-Graduação em Sistemas Aquáticos Tropicais, Universidade Estadual de Santa Cruz, Rodovia Jorge Amado, km 16, 45662-900 Ilhéus, Bahia, Brazil; 4 Departamento de Ciências Biológicas, Universidade Estadual de Feira de Santana, Avenida Transnordestina, CEP44036-900, Feira de Santana, Bahia, Brazil

**Keywords:** Atlantic Forest, bioacoustics, vocalization, Holoadeninae, range extension

## Abstract

Advertisement calls can be used to aid solving taxonomic problems and understanding the evolution of certain groups. In this study, the advertisement call of *Eleutherodactylus bilineatus* is described. It is composed by two different notes with a total duration of 0.529–4.241 seconds and dominant frequency of 1.72–3.45 kHz. Additionally, new data is provided on the geographical distribution of *Eleutherodactylus bilineatus* and the most inland record for this species.

## Introduction

A recent research on the phylogenetic relationships within the anuran clade Terrarana, from the Brazilian Atlantic rainforest frog genus *Ischnocnema*, included *Eleutherodactylus bilineatus* as *incertae sedis*, likely close to the genus *Noblella* and *Barycholos* within the Holoadeninae (Canedo and Haddad 2012). Specimens of this species are small in size (snout–vent length of 20 mm in males and 26 mm in females) and inhabit the leaf litter of moist forests from the southern and central parts of Bahia, northeastern Brazil (Bokermann 1975, Frost 2016). It can be found up to 800 m above sea level and is occasionally encountered in cocoa plantations (Dias et al. 2014a,b, Juncá and Pimenta 2004).

**Figure 1.**
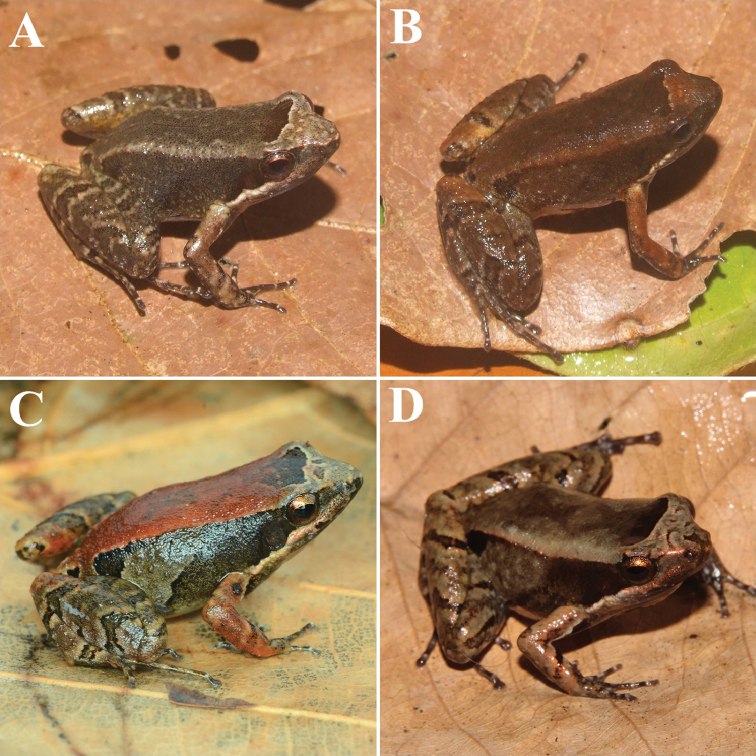
Different individuals of *Eleutherodactylus bilineatus* showing some variation of the dorsal pattern of the species. **A** and **B** RPPN Mata do Passarinho, Macarani **C** Estação Ecológica Wenceslau Guimarães and **D** Serra do Corcovado, Almadina, Bahia, Brazil (photographs A, B and D Iuri R. Dias, C Rafael O. Abreu).

*Eleutherodactylus bilineatus* (Figure 1) has a dark brown dorsal surface with two clear longitudinal stripes on each side of the body which inspire its popular name “Two-lined Robber Frog” (Bokermann 1975). It is not easily detected, and although their reproduction is presumably by direct development (Juncá and Pimenta 2004), there is limited information about its ecology and natural history. Here the advertisement call from two populations of *Eleutherodactylus bilineatus* is described and an updated map of the geographical distribution of this species provided, including new occurrence points.

## Materials and methods

During the execution of the Project “Diversity and genetic structure of the amphibians of the Central Corridor of the Atlantic Forest of southern Bahia” supported by the Boticário Group Foundation for Nature Protection (Project n°.0991_20132), field expeditions were conducted lasting 7-8 days, using the same methodology and similar sampling efforts to nine different locations (Serra da Jibóia, Serra do Timbó, Wenceslau Guimarães, Serra do Corcovado, Pedra Lascada, Serra Bonita, Serra das Lontras, Macarani and PARNA Alto do Cariri), in the Atlantic Forest of Bahia. Specimens deposited in the Museum of Zoology of Universidade Estadual de Santa Cruz were also examined (Table 1), looking for new reports on the distribution of *Eleutherodactylus bilineatus*.

**Table 1.** Distribution records of *Eleutherodactylus bilineatus* based on literature review, museum data (MZUESC) and field works.

Municipality	Localities	Latitude / Longitude	Reference	Voucher analyzed
Almadina	Serra do Corcovado	-14.7011, -39.6625	Dias et al. 2014b	MZUESC 17015
Amargosa	Serra do Timbó	-13.0365, -39.6325	Marciano-Jr et al. 2014	MZUESC 17026-17036
Arataca	Serra das Lontras	-15.1624, -39.3437	This study	MZUESC 17025
Barro Preto	Serra da Pedra Lascada	-14.7723, -39.5408	This study	MZUESC 17016
Boa Nova	–	-14.3591, -40.2383	Berneck et al. 2013	-
Cairu	Fazenda Subaúma	-13.5067, -38.9812	Silvano and Pimenta 2003	-
Camacan	RPPN Serra Bonita	-15.4413, -39.5189	Dias et al. 2014a	MZUESC 8616-17; 8359; 8457
Canavieiras	–	-15.6750, -38.9469	Berneck et al. 2013	-
Guaratinga	Fazenda Vista Bela	-16.4529, -40.0586	Silvano and Pimenta 2003	-
Igrapiúna	Reserva Ecológica da Michelin	-13.8585, -39.1728	Camurugi et al. 2010	MZUESC 14222-14223
Ilhéus	CEPLAC/UESC	-14.7867, -39.2249	Bokermann 1975	MUESC 8110
Jequié	–	-13.9654, -40.0002	This study	MZUESC 7961
Jussari	RPPN Serra do Teimoso	-15.1675, -39.5444	Pimenta and Silvano 2002	-
Macarani	RPPN Mata do Passarinho	-15.7907, -40.5192	This study	MZUESC 16979-16991
Nilo Peçanha	Fazenda São João	-13.6585, -39.1884	Pimenta and Silvano 2002	-
Itarantim	Serra do Mandim	-15.6295, -39.9803	This study	MZUESC 15095-15097, 15855-15856
Santa Teresinha	Serra da Jibóia	-12.7283, -39.5694	Juncá 2006	MZUESC 17007-17014; MZFS 309, 600
Uruçuca	Fazenda Provisão	-14.6512, -39.2232	This study	MZUESC 14444
Valença	RPPN Água Branca	-13.3791, -39.0916	This study	MZUESC 13658
Wenceslau Guimarães	Estação Ecológica de Wenceslau Guimarães	-13.6285, -39.6264	Pimenta and Silvano 2002	MZUESC 17017-17019

The advertisement call of *Eleutherodactylus bilineatus* was recorded at two sites in Bahia state: Serra da Jibóia, Santa Terezinha municipality (-12.728397; -39.569476, 790 m a.s.l.) and RPPN (Private Natural Heritage Reserve) Mata do Passarinho, Macarani municipality (-15.79071; -40.51927, 850 m a.s.l.). In the Serra da Jibóia the recordings were made on three different occasions: December 04, 1995, total of 10 calls from two males (air temperature = 21°C, 19:40h); April 21, 1997, four calls from one male (air temperature 20°C, 20:50h); and March 03, 2015, total of 36 calls from three males (air temperature 21.4°C, 18:15h). The advertisement call (n = 4) of one male from RPPN Mata do Passarinho (encountered in November 27, 2014) is also included in our analysis, recorded after it was placed in a plastic bag. These calls showed the same acoustic patterns as the calls from the males recorded in their natural environment.

Recordings from 1995 and 1997 were made with a SONY WM-D6 Digital Audio Track (DAT) with a directional SONY microphone. For the recordings from 2014 and 2015 a Sennheiser ME45 microphone with a K6 power module connected to a Tascam DR1 digital recorder was used. All recordings were made from a distance of about 40 cm from the frogs. Calls were recorded at a resolution of 16 bit and 48 kHz sampling rate. Waveform and spectrogram were made using Raven Pro 1.4 and analyzed with a Fast Fourier Transformation of 256 points, 50% overlap for an entire call and Window Hamming. For all other configurations the “default” settings of Raven were used. Terminology follows Littlejohn (2001). Voucher specimens are deposited at the Museu de Zoologia da Universidade Estadual de Santa Cruz (MZUESC), under catalog numbers MZUESC 17007-17008 from Serra da Jibóia and MZUESC 16979-16991 (one of these specimens was recorded while specimens were kept in a plastic bag in Macarani) and Museu de Zoologia da Universidade Estadual de Feira de Santana (MZFS), under catalog number MZFS 309 and 600 from Serra da Jibóia.

## Results and discussion

During field expeditions, 151 individuals of *Eleutherodactylus bilineatus* were registered in all locations sampled, except in PARNA do Alto do Cariri, municipality of Guaratinga, where the species was not found. The areas located more northwards revealed the largest abundances of this species: Serra do Timbó (n = 44), Serra da Jibóia (n = 43) and Wenceslau Guimarães (n = 35). In Macarani, located in the southeastern portion of Bahia we also encountered a high abundance with 24 individuals. At the other localities, records were limited to only one or two individuals.

**Figure 2.**
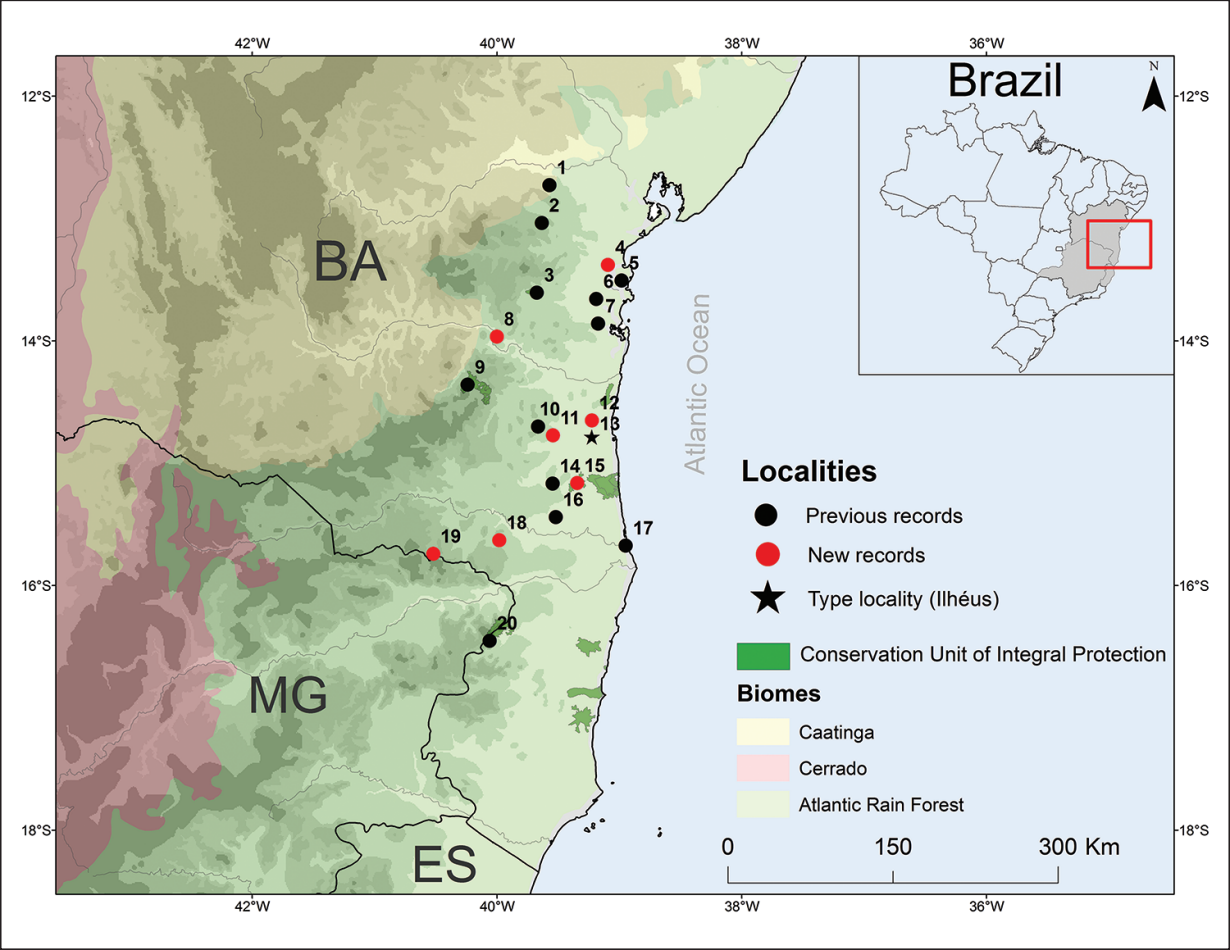
Distribution map of known localities for *Eleutherodactylus bilineatus*. Key: black star = type locality; red circle = new records; black circles = literature records. Municipalities = **1** Santa Teresinha **2** Amargosa **3** Wenceslau Guimarães **4** Valença **5** Cairu **6** Nilo Peçanha **7** Igrapiúna **8** Jequié **9** Boa Nova **10** Almadina **11** Barro Preto **12** Uruçuca **13** Ilhéus (Type Locality) **14** Jussari **15** Arataca, **16** Camacan **17** Canavieiras **18** Itarantim **19** Macarani and **20** Guaratinga. BA = state of Bahia; MG = state of Minas Gerais and ES = state of Espírito Santo. More details of the records are present in Table 1.

Four new distribution records of specimens deposited in the Museum of Zoology of Universidade Estadual de Santa Cruz were found. Thus, the distribution map for the species is updated (Table 1, Figure 2), including seven new records and expanding the known distribution of *Eleutherodactylus bilineatus* in 110 km eastward of the RPPN Serra Bonita, in the municipality of Camacan (Dias et al. 2014a) to the RPPN Mata do Passarinho in the municipality of Macarani, representing the most inland record for the species. Thus, *Eleutherodactylus bilineatus* is distributed in rainforest areas, semideciduous seasonal Forest, and also in shaded cocoa plantations (locally known as “cabrucas”), from the Paraguaçu river to the surroundings of the Jequitinhonha river with its most inland records coming from Boa Nova and Macarani. The species can be found from sea level up to 900 meters.

Males began calling in the early evening, at places with dense leaf litter on the forest floor, near fallen trunks and tabular roots. During our observations (March 03, 2015) several individuals (n = 5–10) were calling together at the sites of recording, and calling activity decreased after 8:00 pm. In the RPPN Mata do Passarinho the advertisement call was heard sporadically, especially during the early evening. However, after one night of heavy rain, several males could be heard calling between 15:00–16:00 h.

**Figure 3.**
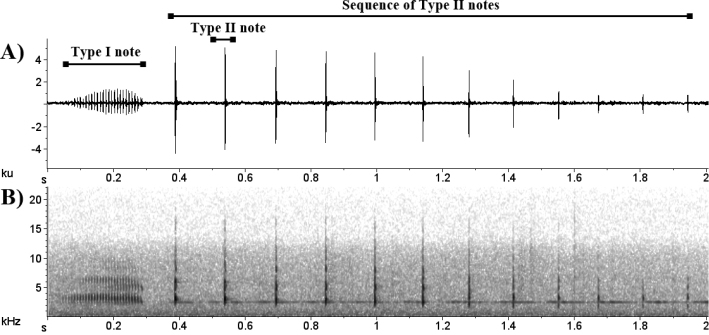
Advertisement call of *Eleutherodactylus bilineatus* (MZFS 600). (**A**) Waveform and (**B**) audiospectrogram. Recorded on April 21, 1997 at 20h 50min. Air temperature during recording 20° C.

The advertisement call (Figure 3) of *Eleutherodactylus bilineatus* had a total duration of 0.529–4.241 s (2.06 ± 0.67, n = 54) and dominant frequency of 1.72–3.45 kHz (2.90 ± 473, n = 54). Two different notes composed the advertisement call; a longer one, here called “Type I” and a shorter one called “Type II”. The two note types are emitted in sequence but in ~15% (n = 8) of the analyzed calls (n = 54) “Type I” was not issued. “Type I” note (or introductory note) had a total duration of 0.124–0.695 s (0.321 ± 0.133, n = 46) and consisted of 17–103 pulses (41 ± 19). The pulse duration of “Type I” note was 0.0035 ± 0.0007 s (0.001–0.006; n = 258) with interval between pulses of 0.005 ± 0.0008 s (0.003-0.007; n = 248). The dominant frequency of “Type I” note was between 2.41–3.27 kHz (3.07 ± 210).

Intervals of 0.07–0.116 s (0.09 ± 0.01, n = 45) separated the two different note types. “Type II” was emitted in a sequence of 6–31 notes (13.98 ± 4.89, n = 54) with duration of 0.001–0.02 s (0.004 ± 0.003, n = 664) each with an interval between notes ranging from 0.07 to 0.21 s (0.13 ± 0.015, n = 660). The dominant frequency of “Type II” notes was 1.72–3.96 kHz (2.91 ± 423) and oscillated between the first (n = 9) and second (n = 31) harmonic. Four harmonic bands could be discerned, with peaks between 1.57–2.07 kHz; the second peak between 2.76–3.45 kHz; the third peak between 4.24–5.02 kHz and the fourth peak between 5.75–614 kHz.

The recordings from Macarani lasted longer and had a higher number of “Type II” notes (more than twice the average) than the recordings from Serra da Jibóia. In Macarani individuals were recorded inside a plastic bag, where they had been placed together with other individuals of *Eleutherodactylus bilineatus*, as well as individuals from other species as *Ischnocnema verrucosa* and *Dendrophryniscus proboscideus*. The observed difference in acoustic parameters could be associated to some kind of social context (e.g. agonistic interactions) and should be further investigated.

The uncertain taxonomic position of this species hampers the comparison of the acoustic parameters with other closely related species. Following Canedo and Haddad (2012) this species would be more closely related with *Noblella* and *Barycholos*. From the 14 known species of these two genera four have had their advertisement calls described (see Table 2): *Barycholos ternetzi* (Lemes et al. 2012); *Noblella carrascoicola* (Köhler 2000), *N. lochites* (Batallas and Brito 2014) and *N. personina* (Harvey et al. 2013). The advertisement call of *B. ternetzi* is a trill consisting of a short multi-pulsed note (30-79 ms) with 4 to 12 pulses per call. The calls of the species belonging to the genus *Noblella* show between 5 and 11 notes lasting between 254–1524 ms with a dominant frequency varying between 3.30-4.39 kHz. The structure of the known calls of the genus *Noblella* is more similar with the “Type II” call of *Eleutherodactylus bilineatus*, with a call composed by series of notes with similar temporal and spectral acoustic parameters. However, none of the compared species showed two types of notes in the same call as in *E. bilineatus*.

**Table 2.** Acoustic parameters of advertisement call of *Noblella* and *Barycholos*, genera more closely related with *Eleutherodactylus bilineatus* according to Canedo and Haddad (2012). Temporal variables in miliseconds (ms).

	*Barycholos ternetzi*	*Noblella carrascoicola*	*Noblella lochites*	*Noblella personina*
Number of Note	1	5–8 (6.0 ± 1.2)	6–8	5–11
Duration of Call	30–79 (49 ± 8)	254–436 (332.3 ± 62.6)	369–537 (428.53 ± 53.60)	570–1524 (1052 ± 307)
Duration of each note	–	12–20	6–17 (11.50 ± 2.90)	13–20 (16 ± 2)
Interval between notes	–	–	51–95 (60.39 ± 5.77)	103–166 (128 ± 14)
Pulses per call	4–12 (7.16 ± 1.47)	–	–	–
Dominant Frequency	3.35–4.31 (3.77 ± 1.75)	3.3–4.0	3.51–3.93 (3.73 ± 0.11)	3.91–4.39 (4.10 ± 0.13)
Reference	Lemes et al. 2012	Köhler 2000	Batalla and Brito 2014^†^	Harvey et al. 2013

^†^ They considered that the call was formed by pulses.

The relationships within this clade (*E. bilineatus*, *Noblella*, *Barycholos*) require a more extensive approach, including morphological information in order to determine the taxonomic position of *E. bilineatus*, as well as the inclusion of molecular data into the phylogenetic analyses of other species of Holoadeninae (Canedo and Haddad 2012). As highlighted by Padial et al. (2014) the relationships within this subfamily provide insights on the possible connection between the Andes (*Noblella*), the Atlantic Forest of Northeastern Brazil (*E. bilineatus*), and the Cerrado (*Barycholos ternetzi*) deserving a more accurate exploration of the biogeography in the future.
